# Investigating the Role of State and Local Health Departments in Addressing Public Health Concerns Related to Industrial Food Animal Production Sites

**DOI:** 10.1371/journal.pone.0054720

**Published:** 2013-01-30

**Authors:** Jillian P. Fry, Linnea I. Laestadius, Clare Grechis, Keeve E. Nachman, Roni A. Neff

**Affiliations:** 1 Department of Environmental Health Sciences, Johns Hopkins Bloomberg School of Public Health, Baltimore, Maryland, United States of America; 2 Center for a Livable Future, Johns Hopkins Bloomberg School of Public Health, Baltimore, Maryland, United States of America; 3 Department of Health Policy & Management, Johns Hopkins Bloomberg School of Public Health, Baltimore, Maryland, United States of America; Public Health Agency of Barcelona, Spain

## Abstract

**Objectives:**

Evidence of community health concerns stemming from industrial food animal production (IFAP) facilities continues to accumulate. This study examined the role of local and state health departments in responding to and preventing community-driven concerns associated with IFAP.

**Methods:**

We conducted semi-structured qualitative interviews with state and county health department staff and community members in eight states with high densities or rapid growth of IFAP operations. We investigated the extent to which health concerns associated with IFAP sites are reported to health departments, the nature of health departments’ responses, and barriers to involvement.

**Results:**

Health departments’ roles in these matters are limited by political barriers, lack of jurisdiction, and finite resources, expertise, and staff. Community members reported difficulties in engaging health departments on these issues.

**Conclusions:**

Our investigation suggests that health departments frequently lack resources or jurisdiction to respond to health concerns related to IFAP sites, resulting in limited engagement. Since agencies with jurisdiction over IFAP frequently lack a health focus, increased health department engagement may better protect public health.

## Introduction

**Figure 1:**
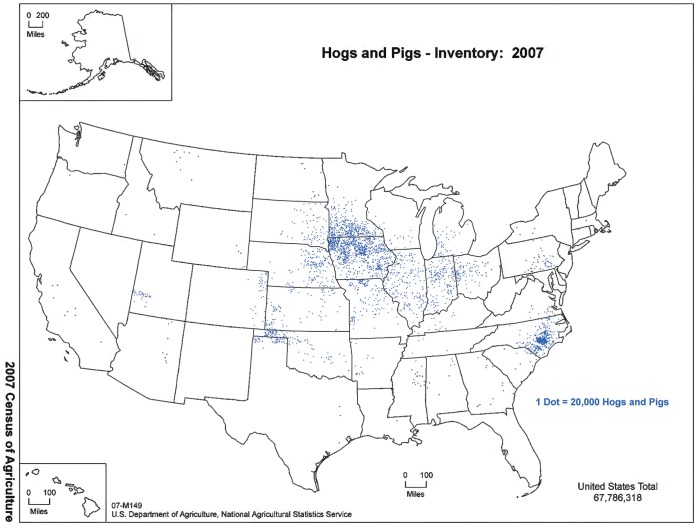
Geographic concentration of hog and pig production in the US. Figure Legend: 2007 Census of Agriculture data from the United States Department of Agriculture (USDA) shows the geographic concentration of hog and pig production in the US. Source: USDA Census of Agriculture; 2007 Census Ag Atlas Maps www.agcensus.usda.gov/Publications/2007/Online_Highlights/Ag_Atlas_Maps/.

Industrial food animal production (IFAP), the dominant meat, dairy, and egg production method in the US, involves housing large numbers of animals in close quarters [1]. Between 1959 and 2007, mean farm size increased over 2,300 percent for hogs and over 30,000 percent for broilers (chickens sold for meat). During this time, the number of farms in the US with swine and broilers decreased by 95.9 and 98.5 percent, respectively [2], [3]. In addition to a dramatic shift to fewer, larger operations, IFAP sites are geographically concentrated, as Figure 1 illustrates for US hog production. The impacts of IFAP are much more widespread than the map indicates, however, because other types of IFAP are concentrated in different areas of the country. Producing large numbers of animals in close proximity results in the concentration of massive amounts of animal waste in small geographic areas, leading to practices such as over-application to fields as fertilizer, storing waste in sheds or large cesspits or lagoons, and transporting waste from overburdened regions [4], [5]. Regulations exist to prevent animal waste from entering surface water, but the US Environmental Protection Agency has noted that many facilities are failing to comply with regulations [6]. Also, there is much variation at the state level regarding regulatory stringency and enforcement [7], [8]. The regulatory term Concentrated Animal Feeding Operation (CAFO) is used to describe these facilities, but we use the term IFAP, which includes CAFOs, as there is concern that sites that use these methods but fail to meet the technical CAFO definition due to size and/or structure can still contribute to serious environmental public health problems.

Our study seeks to understand the role of state and local health departments (HDs) in addressing citizen health concerns related to IFAP facilities in the US. We also explore potential barriers that may limit agency activities aimed at addressing these issues.

Documented air emissions from IFAP include hydrogen sulfide [9], particulate matter [9], endotoxins [10], ammonia [11], allergens [12], and volatile organic compounds [13], [14]. Exposure to these emissions has been associated with multiple respiratory, cardiovascular, and neurological outcomes for IFAP workers [15], [16]. Community health effects largely parallel those from occupational exposure, with growing evidence suggesting that residential proximity to IFAP facilities also increases respiratory health risks [13], [17]–[19]. Studies link chronic exposure to animal production odors to headaches, nausea, upset stomach, mood disorders, and sleep problems [20]–[23]. Water is also a concern; rural residents who rely on private wells for their water supply are at particular risk as animal waste contaminants, including nitrates, pathogens, pharmaceuticals, metals, and hormones, can leach into ground water [24], [25].

Studies in two states have shown that IFAP facilities are disproportionately sited in low-income communities with high-percentage minority populations [26]–[28]. These populations are at increased risk of respiratory conditions such as asthma, and are already significantly less likely to have health insurance than higher-income white populations [29], [30], thus increasing both their vulnerability to the health consequences of IFAP pollution and the challenge of obtaining medical advice and treatment.

In some cases, HDs have funded or performed investigations to collect data on IFAP health effects [28], [31]–[34]. On the other hand, two of the authors (RN and JF) witnessed an individual at a public meeting state his belief that the health department would surely know about any health concerns posed by an IFAP facility and alert the public to the situation. This was striking because in this case the HD had little to no involvement in the decision to permit the facility in question, nor with monitoring or regulation of IFAP sites in the area. This led to the realization that research was needed to examine the extent to which human health concerns associated with IFAP are reported to HDs, and how agencies with IFAP operations in their jurisdiction respond to public concerns about health problems that may be caused by these operations.

Environmental health concerns related to air and water quality have long been key to the conception of public health in the US [35]. The 10 essential public health services, which include monitoring, diagnosing and investigating community health hazards and informing/educating community members, suggest that some aspects of IFAP would fall within public health agency mandates [36]. Environmental health responsibilities, however, are increasingly shared between Departments of Health and Departments of Environment, resulting in challenges to coordination and data sharing [37]. A report by the National Council of State Legislatures indicates that primary state-level regulatory authority over IFAP facilities falls largely with Departments of Environment and Natural Resources and that health departments generally have little or no role in these activities [8], which raises concerns that regulations may not be designed to protect human health.

Another critical factor to consider when examining the HD role in addressing health impacts from IFAP is that many local and state HDs in the US have suffered significant and sustained resource reductions, in part attributable to the recession and weak economic recovery in the early 2000s [38], [39]. Limited staff and resources hamper state HDs’ ability to investigate non-communicable disease clusters [40]. Additionally, lower rates of illness and better health outcomes have been linked to more comprehensive HD programs and higher levels of resources, including funding and staff [41]–[44]. Identifying and publicizing the implications of HD resource levels and program characteristics is important to effectively addressing the limitations and gaps that may be experienced by HD programs.

## Methods

Due to the exploratory nature of the research, we determined that a qualitative approach to data collection and analysis was most appropriate. We performed semi-structured interviews with state- and county-level HD employees in eight states, and with a community member in each of those states. We used USDA Census of Agriculture hog inventory data to rank all US counties in two ways: 1) 2007 county hog inventory at operations with 1,000 or more hogs, and 2) increase in hog inventory at 1,000+ head operations between 2002 and 2007. Many hogs produced in intensive settings or a large increase in the number of hogs may lead community members or others to request that HDs become involved with IFAP. We sought to increase the chance that the relevant hog operations were near residential areas by ranking the top sixty counties from each list by population density using 2000 Census data. We selected the top fifteen counties by population density from the two lists and, due to overlap, eighteen counties in eight states remained. To ensure that no state was disproportionately represented, we limited the sample to a maximum of two counties per state. The final sample comprised fourteen counties in eight states.

To identify appropriate HD personnel to interview, we performed background research using health department websites and contact with the Association of State and Territorial Health Officials (ASTHO). We contacted HD staff who led environmental health divisions within an HD or the head of the HD if there was no division focused on environmental health. At times, HD staff we contacted referred us to others in the department because they could better answer our questions. In two instances, we found that the county HD did not address environmental health concerns. In these cases, we included the county level department that handled environmental health concerns, generally an environmental services department, instead of the HD. To avoid unnecessary complication, we include these agencies with the county HDs in the results except where they differed substantially.

We identified community members through Internet research on involved organizations in each state and by asking known contacts who work on IFAP issues for suggestions. Recognizing that speaking to only one community member per state was limiting, we nonetheless felt it important to complement HD comments with community perspectives. We sought affected or potentially affected residents who had been active in addressing IFAP issues in their state, as opposed to lawyers, environmental scientists, or others who were involved professionally but not directly affected by IFAP operations where they live. When possible, we interviewed community members who were from the included counties. As these individuals were often members of community groups working on IFAP concerns, they spoke both as individuals and as representatives of their groups. We conducted all interviews by telephone.

The questionnaire included mostly open-ended and some closed-ended questions (See Appendix S1 for survey instruments). We sent background questions to HD staff members prior to the interview so information on budgets and workforce could be compiled beforehand. We did not use the terms IFAP or CAFO in the interviews. Before beginning each interview, we read participants a confidentiality statement. The Johns Hopkins School of Public Health Institutional Review Board (IRB) determined the study was exempt from IRB oversight.

After data collection was complete, notes from six interviews were double coded by JF and LL through an inductive coding process using *HyperRESEARCH 3.0.3* (ResearchWare, Randolph, MA). We then jointly discussed codes, developed a uniform codebook, and applied it to the remaining interviews. Due to the qualitative nature of the study and small, non-representative sample, we provide limited numeric information from the data to avoid giving the impression that the findings are generalizable to a larger population [45].

## Results

We conducted telephone interviews with staff from thirteen county HDs and eight state HDs, plus eight community members between November 2010 and January 2012. One county HD contacted declined to participate.

### Health Departments

#### Contacts made by community members

HD staff reported receiving calls about IFAP facilities generally a few times a year to a few times a month, with some reporting that they have not been contacted about issues related to IFAP. We read participants a list of the most common health concerns associated with IFAP, as identified in the literature. Of these, the most common reasons for calls were odor, water quality concerns, respiratory health, general health concerns, and stress. Individual residents, rather than physicians or organized groups, usually made these calls. Almost all HD staff noted that the frequency of related calls had either remained constant or decreased over the past few years. A few county HD staff cited the passage and/or enforcement of ordinances and regulations that limited new facility construction or improved waste management practices as an explanation for the reduction in call volume. One county HD staff member noted that the volume of reports changed “when the state actually started enforcing feedlot rules.” Two county-level HD staffers in the same state indicated that the number had increased in recent years, and some state HD staff members said call volume generally increased when new facilities were being proposed or built.

#### Response to calls

Overall, staff indicated that HDs do not have a prescribed response to reported concerns about IFAP facilities, and most counties and states described their response as a case-by-case process. Few HD staff reported keeping formal records of calls. In almost all instances, regardless of the type of concern, county and state HD staff reported that they refer residents with concerns about IFAP to another agency (Table 1). Most indicated that this was due to their agencies’ lack of regulatory authority over animal production farms. Specific referrals generally depended on what agency had regulatory authority over IFAP in that particular state, with referrals to Departments of Environment and Natural Resources most common. Staff from a few counties and states said they might speak to the IFAP facility operator, but noted that they could only request voluntary changes. A state HD staff member noted, “the best we can do is bring it to the operator’s attention and hope they take care of it voluntarily,” and a county HD staff member said, “we have no control over manure spreading or manure management, all we can do is consult with the farmer to try to work with them.”

**Table 1:** Referrals reported by State and County Health Department staff in response to concerns related to IFAP.

State Departments of Agriculture
Soil Conservation Districts
Cooperative Extensions
State Departments of Environment/Natural Resources/Pollution Control
State HD referral to county HD and vice versa

#### Additional involvement

We also queried HD staff about their involvement with IFAP issues more generally. Staff from eight counties and three states reported little to no involvement; others’ activities are described below.

About half of the county and state HD staff reported that their agencies collaborated with other agencies and government entities at times on IFAP issues, for example through communicating with them or visiting problematic IFAP facilities along with other agencies if the situation warranted their attention. Most reported that they had not engaged with citizen groups active on IFAP issues, and that they were not aware of any such citizen groups in their regions.

Most county and state interviewees reported no involvement in decision-making regarding animal agriculture. A small number said they had been involved with regulatory issues such as zoning, siting requirements, and setback requirements (distance between a building and the property edge). A few state HD staff members said they serve on advisory committees or provide input to those making decisions. Only two county agency staff members mentioned involvement with permitting IFAP facilities; these counties were atypical in that their environmental health programs were located within their environmental services departments rather than HDs.

About half the states reported collecting some IFAP-relevant data in the past (e.g. well testing, counting flies, air monitoring). These instances appeared to have been rare, however, and primarily motivated by IFAP facilities with particularly poor standards rather than routine data collection. Additionally, the vast majority of county and state HD staff interviewed had not provided any educational services to residents regarding potential health effects of animal agriculture. Efforts mentioned by staff that did provide information included informally explaining IFAP health effects to community members and creating an online document reviewing research and opinions on IFAP and health.

#### Barriers to action

State and county HD staffers reported multiple barriers to addressing concerns about intensive animal agriculture. Lack of jurisdiction over IFAP was the most frequently reported. In almost all states and counties examined, odor and air quality issues were reported to be outside the jurisdiction not only of HDs, but also of other state and county agencies, including Departments of Environment and Agriculture, due to a lack of regulation. One state has an ordinance pertaining to hydrogen sulfide, and the interviewee said they are often contacted by residents in other states asking what they can do about air quality problems because those states do not have air quality regulations relevant to IFAP emissions. Some interviewees also mentioned resource and infrastructure challenges. More than half of the state HD staff mentioned these as preventing them from undertaking a formal study to assess an issue like well water quality, and one county stated that they are trying hard enough to handle their own mandated programs with limited resources. In addition, HD flexibility in allocating environmental health funds varied. About half of HD staff interviewed indicated that they had no or quite limited flexibility in allocating these funds. No HD received funds specifically designated for supporting IFAP related efforts or to train staff in this area. Levels of expertise also hindered response, with one state HD staffer noting that they simply do not have the expertise needed to have their own regulatory program on IFAP. Another state HD staff member felt the current science on IFAP and public health is insufficient and does not justify health agencies providing guidance on IFAP for other agencies. A few county HD staff members stated that they do not believe that odor is associated with health effects, and one county HD staff member mentioned that the agency with regulatory authority over IFAP will investigate spills, but “if it’s just smell, they don’t worry about it.”

Many HD staffers at both the state and county level also described political barriers to addressing the issue, including industry political influence in the state or county, agriculture’s importance for economies in rural areas, and intentional efforts by legislators and administrators to avoid enforcing IFAP regulations. A county HD staffer explained that their early efforts to pass an ordinance to regulate IFAP facilities failed in part because so many people in the area make an income from farming. Another respondent said a state law had been changed to thwart local efforts to control CAFO siting, invoking the strategy of preemption, and a county-level interviewee described state legislators pressuring agencies to avoid looking into the issue. Several state HD staff members indicated that their state governments feel that the financial incentive to have large farms outweighs other concerns, and one noted that they had to be extremely careful with any statements they make on the issue of IFAP and health because anything perceived to be detrimental to industry could result in a lawsuit. Another noted that his department has to “protect health while still allowing breathing space for [industry] to ‘make a buck’.”

#### Needed resources

We provided HD staff with a list of possible resource needs and asked them to indicate any they needed to more effectively address health concerns stemming from IFAP (Table 2). The most commonly indicated need was for educational materials that could be distributed to the public on possible health effects of IFAP facilities. A few county HD staff members indicated that they had no need for any additional resources, primarily because this was not an issue they planned to address.

**Table 2:** Resources indicated by HD staff as needed to address IFAP concerns (ranked by number of HDs indicating need).

1. Educational materials for distribution
2. Increased funding for department
3. Training for staff on issues relevant to animal production farms
4. Updated information from researchers on health effects of concern
5. Environmental quality tracking tools
6. More staff
7. Different political climate
8. Funding specifically for animal production farm activities
9. Connections to experts

### Community Members

All community members interviewed expressed concerns about IFAP-related health impacts. Community members reported a wide range of activities, both personally and with community groups, to address IFAP concerns (Table 3), including contacting local and state HDs and local boards of health. As Table 3 shows, community members and organizations have been assuming roles that would traditionally fall under HD purview, including environmental monitoring, educating and providing information to the community, seeking information from other government agencies, and partnering with researchers. Communication with other local and national level groups addressing IFAP was common.

**Table 3:** Actions taken by community members/groups in response to IFAP concerns.

Contacting local and state agencies (departments of health, environment, natural resources, and agriculture), state legislators, and governors
Forming community organizations and seeking advice from other organizations that have been working on this issue
Holding public events and conducting public education
Reading scientific literature on IFAP and health
Organizing meetings
Attending permit hearings and reviewing permit applications
Conducting own environmental monitoring
Requesting samples to be taken
Media advocacy
Advocating for state legislation
Forming a group to hire a lawyer
Contacting local police
Collaborating with researchers
Collaboration with other national and regional groups
Serving on local boards (e.g. board of health)

No community members reported having had an interaction with an HD about an IFAP issue that resolved their concerns. Generally they stated that HDs were not engaged with this issue. Some community respondents indicated that HD staff had attended meetings on this topic or showed an initially encouraging response; however, they said that the HDs ultimately took no action. One community member thought that the HD was placing the burden of proof on community members. Community members who said they were unsuccessful in having their concerns addressed by contacting HDs generally did not persist in contacting them, and instead moved on to other agencies and approaches. Despite this, almost all community members interviewed indicated that there should be a role for HDs in addressing this issue complementary to that of permitting agencies.

Community interviewees stated that the number of concerns reported to agencies was likely limited by agriculture’s influence in small rural communities. One suggested that everyone in the county is related to someone who works in agriculture and asked, “Who is willing to rat out their cousin?” Another described social stigma associated with expressing concerns about IFAP and noted several instances of harassment against individuals who had done so.

Community members recognized many of the themes expressed by HD staff regarding barriers to HD response. They stated that perceived barriers included politics and animal agriculture’s economic importance, limited agency resources, and active efforts by agricultural interests to minimize regulatory attention to IFAP facilities. Many community members perceived an inappropriate connection between government agencies, legislators, and industry. One community member noted that “they’re not even working for us anymore; it’s like they’re working for industry.” Another community member suggested that county HD staff members are privately very concerned about IFAP and health, but publicly refrain from addressing it due to “political posturing.”

## Discussion

Evidence suggests that contaminated air, soil, and water near IFAP facilities can pose significant public health threats. Despite that, this analysis found that the sampled HDs received few contacts from the public on IFAP issues, and engaged little on the topic. Significant barriers to engagement were identified.

The low call volume to HDs should probably not be interpreted as a lack of public concern, but rather as a function of the unique political and social circumstances surrounding IFAP in many rural communities. Community members commented that when they call HDs and are referred to other agencies because animal agriculture is said not to be under the HDs’ jurisdiction, they often stop contacting the HD about IFAP concerns. Community members also reported social pressures that appear to limit the voicing of IFAP-related concerns.

Due to regulations relevant to IFAP, jurisdiction is almost exclusively within Departments of Agriculture and Environment/Natural Resources. As human health is generally not a primary part of the mission of these agencies, if it is included at all, HD involvement even in an advisory capacity can be valuable. Unfortunately, many barriers in addition to jurisdiction are currently preventing HDs from engaging on this issue, including resource constraints, lack of expertise, and political pressure.

This is the first study to examine formally the role of HDs in responding to community concerns arising from IFAP operations. We used qualitative methods to engage with both HD staff and community members to gain context regarding their respective views on roles, responsibilities, and responsiveness. Due to the use of a small sample that may not be representative of all US regions impacted by IFAP we cannot extrapolate findings to all counties and states with these types of operations. Additionally, the opinions and perceptions of the individual HD staff members interviewed likely influenced results. While there was frequently only one staff member identified as relevant at each HD, it is possible that other staffers may have provided substantively different responses. Despite these considerations, we anticipate that the results of our investigation have identified common themes in agricultural communities.

For community members in particular, the sample was limited and likely does not reflect the full range of views within the surveyed regions. Views identified here may not, for example, be representative of the views of rural community members who have not actively sought to address IFAP issues. Additionally, inclusion of others involved with these issues, such as staff at nonprofit environmental organizations instead of community members, would likely result in different responses. Despite this, we believe their inclusion adds further context that aids in understanding the perspectives of HD staff on issues related to IFAP operations.

### Conclusion

It is critical for affected communities, healthcare providers, policy makers, and public health professionals to be aware of the limited engagement on this issue by HDs. We found that HD staff reported inadequate resources and/or jurisdictional authority needed to monitor and regulate IFAP facilities; as a result, IFAP facilities that pose a threat to public health may go unnoticed and unaddressed by HDs. While giving HDs a formal role has the potential to improve public health protection, HDs could play a more significant role even with no change in jurisdictional authority if resources and the political landscape changed. HDs with IFAP operations in their county or state should be provided with resources such as training, educational materials, and increased funding. These resources could be provided through organizations working on IFAP and public health issues, state legislatures, or from the federal government as part of the national oversight of CAFO permitting programs. It should be noted, however, that even with these resources or a change in jurisdictional authority, political barriers will likely remain a significant challenge to fully addressing IFAP and public health.

There are many potential directions for future research that can build on these findings. For example, a questionnaire informed by these results could be developed that would allow researchers to study a much larger sample of HD staff and/or community members. A larger study would facilitate more robust comparisons between states and regions and also allow investigators to explore additional factors such as which characteristics increase the likelihood that an HD will take on a more proactive role or how race, ethnicity, or socio-economic status of affected communities impact these situations. Other groups, such as staff of agencies with jurisdiction over IFAP and environmental organizations, could also be included. We hope this study will serve to include IFAP in the wider debate on resource limitations faced by HDs and their role in protecting environmental public health and spur additional research on this topic.

## Supporting Information

Appendix S1 Health Department Personnel and Community Members Questionnaires.
(DOC)
