# Supplementary material for: Investigating the Role of State and Local Health Departments in Addressing Public Health Concerns Related to Industrial Food Animal Production Sites
Source: PLoS One. 2013 Jan 30;8(1):e54720. doi: 10.1371/journal.pone.0054720 (PMC3559890; doi:10.1371/journal.pone.0054720)
Supplement: Appendix S1 — Health Department Personnel and Community Members Questionnaires. (DOC) [file pone.0054720.s001.doc]

Appendix S1: Health Department Personnel and Community Members Questionnaires

**1. County and State Health Department Personnel Questionnaire**

*The first seven questions were sent ahead of time so information could be collected.*

*The following set of background questions provides information regarding the structure, size and scope of the health department’s activities.*

Workforce:
1. How many FTE staff work at the health department?

2. How many FTE staff within the department focus on environmental health (using the definition below)?

“Environmental health comprises those aspects of human health, including quality of life, that are determined by interaction with physical, biological, and social factors in the environment. It also refers to the theory and practice of assessing, correcting, and preventing those factors in the environment that may adversely affect the health of present and future generations.”

Funding:
3. Which of the following describes the main funding source(s) of environmental health within the health department?  (Provide approximate percentages if possible.)

a. State    ___ %

b. Federal pass through ___ %

c. Federal direct  ___ %

d. Medicaid   ___ %

e. Medicare   ___ %

f. Fees    ___ %

g. Other (please describe) ___ %

4. What is the overall budget of the health department?

5. What proportion of the health department’s budget is used for environmental health programs and services (estimate if needed)?

6. To what extent does your agency have flexibility in how you allocate environmental health funds versus funds being allocated to specific programs?

7. Over the last five years, has the funding for environmental health in your state gotten better, worse, or stayed the same?


Confidentiality Statement
Your personal information will be kept confidential.  Your name and position will not be reported in the results of this study, and your responses will not be linked to your state.  Your personal information and responses will be kept on password-protected computers and will not be shared outside of the research team.

1. What is your position at the health department?

2. How many years have you been working at the department?

3. What education level have you completed?

4. How many years have you lived and/or worked in the state?

5. To your knowledge, has the health department been contacted by the public about health concerns that people were associating with living or spending time near animal production farms or manure?

6. If contacted, approximately how many times in a typical year?

6a. Has this changed over time and if so, how?

6b. Are the contacts you receive about this issue spread out across the state, or are they         mainly coming from certain regions?

6c. How does the number of contacts you receive about animal production farms compare to the numbers you receive regarding other land uses, such as industrial sites?

7. To your knowledge, who made these contacts?  (Choose all that apply.)
*(Do not supply list/examples to interviewee unless they ask for clarification.)*

a. Individual describing own concerns

b. Health care provider

c. Members of an organized campaign

d. Other (please describe)

8. What concerns were described? (Choose all that apply.)
*(Do not supply list/examples to interviewee unless they ask for clarification.)*

a. Effects of odor

b. Respiratory health

c. General health

d. Ground water quality/contaminated well

e. Waste getting on property

f. Violations of regulations

g. Neurological conditions

h. Infections

i. Headaches

j. Stress

k. Other (please describe)

9. Who (what position) within the agency is tasked with responding to such concerns?

10. Is there a set process in your department for responding to complaints about animal production farms, or are they handled on a case-by-case basis?

11. What is usually done in response to a complaint?
*If a referral to another Department is mentioned, ask who specifically they refer it to, and/or what section of the department.*

11a. So, just as an example, what would you do if someone called your department to complain about headaches associated with odor coming from an animal production facility? (Only ask if not redundant)

12. Are records kept of complaints and responses?

13. In your jurisdiction, do health departments (local or state) play a formal role in addressing health concerns associated with animal production farms?

13a. In our previous work on this issue, we have heard residents say:

*“If animal production farms were causing health problems, then the Health Department would know that and do something about it.”*

Do you feel that this sentiment is an accurate description of how this issue is handled in your state?

13b. Do you think health departments should play a formal role in animal production farm issues to address health concerns?

13c. What role(s) do you think is appropriate for health departments regarding animal production farms?

14. Has your department carried out any health education activities relevant to concerns about health effects associated with animal production farms?  If so, please describe.

15. Has your department conducted surveillance or collected any data relevant to animal production farms?  Information collected could include: private well testing, air/water monitoring, symptoms experienced by residents, etc.

a. If so, what type of data was collected?

b. Were the locations of animal operations or manure spray fields taken into account when data were collected or analyzed?

c. Has this information been written up in a report or distributed in some way?

16. Has your department worked with any local, state or federal agencies to respond to health concerns about animal production farms?  If so, please describe.

17. To your knowledge, are there any organizations or groups of citizens in your state that work on local animal production farm issues?  If so, has your department had contact with or ever worked with them?  Please describe.

18. Has your department ever been engaged with decision-making processes, such as siting, permitting requirements or fines, regarding animal production farms?  Activities could include participating in or observing meetings, participating on an advisory board, or providing input to those engaged in decision-making.  If so, please describe.

19. Are there any additional actions your department has considered, but has not taken due to financial, political, capacity or other barriers?  (Please describe action that was considered and barrier.)

20. Does your department need certain resources or different circumstances to enable you to more effectively address health concerns associated with having animal production farms in your state?  Please describe.
*(Leave question open-ended and then present list.)*

a. More staff dedicated to environmental health

b. Increased funding for environmental health

c. Funding specifically for animal production farm activities

d. Training for staff on public health issues relevant to animal production farms

e. Different political climate

f. Connections to experts

g. Updated information from researchers on health effects of concern

h. Educational materials for distribution

i. Environmental quality tracking tools

j. Other (please describe)

20a. *If they mention a different political climate AND other resources:*

If the political climate stayed the same as it is now, how would you make use of the other resources?

21. We are also contacting state Departments of Agriculture as a part of this study. Is there anyone in particular who works on animal production farm issues that you suggest we contact in your state?

22. Is there anything else you would like to add that you did not have an opportunity to share?

Thank you for participating!

**2. Community Member Questionnaire**

# Confidentiality Statement

# Your personal information will be kept confidential.  Your name and position will not be reported in the results of this study, and your responses will not be linked to your state.  Your personal information and responses will be kept on password-protected computers and will not be shared outside of the research team.

1. How many years have you lived in the area? What county do you live in?

2.  Are you a member of an organized group working on such issues related to animal production farms?  If so, what is the name of the organization and what is your role?

3. What main activities do you or your organization carry out to address concerns about animal production farms in the area?

4. Do you think animal production farms pose public health concerns?

           a. What problems do you worry about for people who live or spend time near animal             production farms and/or manure spray fields?

           b. What information or experiences formed these concerns?

5. What government agencies have you or others contacted about possible health concerns linked with living or spending time near animal production farms or manure?  (i.e. your state or local health department, the agency that is responsible for animal production farm permits, a local Board of Health, elected officials)

a. How many times?

           b. What concerns did you talk about?

           c. To your knowledge, how were such instances handled?
 
6. Have government agencies ever worked with or met with you or another citizen group in an effort to address animal production farm issues?  Please describe.

7. Have any government agencies collected data relevant to animal production farms in your area, such as air/water monitoring or symptoms experienced by residents?
           a. If yes, what type of data was collected?
            
           b. Has this information been analyzed and/or shared with the public?
            
8. On a scale of 1 to 10, with 1 being not a concern at all and 10 being a top priority, to what extent do your county and state health departments seem to be concerned about potential public health effects of animal production farms in your area?
           County:
           State:

           Do you think the departments’ levels of concern are appropriate?
           County:
           State:

9. To your knowledge, has the health department (county or state) carried out any health education activities relevant to concerns about living near animal production farms?  If so, please describe.
           County:
           State:

10. To your knowledge, has the county or state health department ever been engaged in decision-making processes regarding animal production farms, including as a participant or observer attending meetings, participation on an advisory board, providing input to those engaged in decision-making, etc (i.e. permit issues)?  If so, please describe this involvement.

11. Do you think health departments (county or state) should play a formal role in animal production farm issues to address health concerns?  Why or why not?

If yes, what role(s) do you think would be appropriate for health departments?

12. Are there any other activities you are aware of that the county or state health department has performed to address potential public health issues relevant to animal production farms in your area?

13. *(If concerned about public health and animal production farms.)*  How effectively do you think the county or state health department is responding to concerns about animal production farms in the area?

*(If they think they are not effectively responding)* What do you think the main barriers are that prevent the health department from more fully responding?

14. Has any other government agency or organization responded to health concerns about animal production farms in your area?  If so, please describe.

15. Is there anything else you would like to add that you did not have an opportunity to share?

Thank you for participating!
